# Baseline microvessel density predicts response to neoadjuvant bevacizumab treatment of locally advanced breast cancer

**DOI:** 10.1038/s41598-021-81914-0

**Published:** 2021-02-09

**Authors:** Kristi Krüger, Laxmi Silwal-Pandit, Elisabeth Wik, Oddbjørn Straume, Ingunn M. Stefansson, Elin Borgen, Øystein Garred, Bjørn Naume, Olav Engebraaten, Lars A. Akslen

**Affiliations:** 1 Centre for Cancer Biomarkers CCBIO, Department of Clinical Medicine, Section for Pathology, Haukeland University Hospital, University of Bergen, Bergen, Norway; 2 Department of Cancer Genetics, Institute for Cancer Research, Division of Cancer Medicine, Surgery and Transplantation, Oslo University Hospital Radiumhospitalet, Oslo, Norway; 3 Department of Pathology, Haukeland University Hospital, Bergen, Norway; 4 Department of Oncology, Haukeland University Hospital, Bergen, Norway; 5 Department of Pathology, Oslo University Hospital, Oslo, Norway; 6 Department of Oncology, Division of Cancer Medicine, Oslo University Hospital, Oslo, Norway; 7 Institute for Clinical Medicine, University of Oslo, Oslo, Norway; 8 Centre for Cancer Biomarkers CCBIO, Department of Clinical Science, Section for Oncology, University of Bergen, Bergen, Norway

**Keywords:** Breast cancer, Cancer microenvironment, Cancer therapy, Tumour angiogenesis, Tumour biomarkers

## Abstract

A subset of breast cancer patients benefits from preoperative bevacizumab and chemotherapy, but validated predictive biomarkers are lacking. Here, we aimed to evaluate tissue-based angiogenesis markers for potential predictive value regarding response to neoadjuvant bevacizumab treatment in breast cancer. In this randomized 1:1 phase II clinical trial, 132 patients with large or locally advanced HER2-negative tumors received chemotherapy ± bevacizumab. Dual Factor VIII/Ki-67 immunohistochemical staining was performed on core needle biopsies at baseline and week 12. Microvessel density (MVD), proliferative microvessel density (pMVD; Factor VIII/Ki-67 co-expression), glomeruloid microvascular proliferation (GMP), and a gene expression angiogenesis signature score, were studied in relation to pathologic complete response (pCR), clinico-pathologic features and intrinsic molecular subtype. We found that high baseline MVD (by median) significantly predicted pCR in the bevacizumab-arm (odds ratio 4.9, *P* = 0.012). High pMVD, presence of GMP, and the angiogenesis signature score did not predict pCR, but were associated with basal-like (*P* ≤ 0.009) and triple negative phenotypes (*P* ≤ 0.041). pMVD and GMP did also associate with high-grade tumors (*P* ≤ 0.048). To conclude, high baseline MVD significantly predicted response to bevacizumab treatment. In contrast, pMVD, GMP, and the angiogenesis signature score, did not predict response, but associated with aggressive tumor features, including basal-like and triple-negative phenotypes.

## Introduction

Angiogenesis, new blood vessel growth from the existing vasculature^1,2^, is a hallmark of cancer and promotes tumor progression and metastasis^1,3^. Bevacizumab is a humanized monoclonal antibody against vascular endothelial growth factor A (VEGF-A), a key factor in this process^4^. It was approved by the FDA in 2008^4^ for use in metastatic HER2-negative breast cancer in combination with paclitaxel^5^, although later studies reported only small improvements in progression-free survival^6,7^ and no effect on overall survival^5–7^. Consequently, the FDA-approval was withdrawn in 2011^4^. In the preoperative setting, however, bevacizumab in combination with chemotherapy has shown improved pathologic complete response (pCR) in several clinical trials in HER2-negative breast cancer^8–12^, with improved overall survival in one study^13^. Currently, although bevacizumab is not routinely used in breast cancer, it has an important clinical role in several other solid cancer types^14^. Also, new combinations including bevacizumab and immune check point inhibitors show promising results^15,16^. A major challenge concerning bevacizumab treatment is the lack of biomarkers for patient stratification^17^.

In tumor tissue, microvessel density (MVD) has shown prognostic value in breast cancer^18,19^. As a novel marker of activated angiogenesis, proliferation of microvessels, pMVD (proliferative microvessel density) has shown even stronger association with prognosis compared with MVD^20^. Other tissue markers of angiogenesis are the “vascular nest” phenotype of glomeruloid microvascular proliferation (GMP)^21^, assumed to be VEGF-A associated^22^, as well as a microarray-based 32-gene angiogenesis signature^23,24^. Presence of GMP has been associated with reduced breast cancer survival and lack of response to chemotherapy^25,26^.

Here, in a phase II clinical trial, patients with locally advanced (HER2-negative) or large breast cancers (≥ 2.5 cm) were randomized to receive chemotherapy ± bevacizumab. The purpose of the study was to evaluate whether tissue-based markers of angiogenesis (MVD, pMVD, GMP, 32-gene signature) could predict response to bevacizumab treatment and provide a basis for patient stratification.

## Results

### High microvessel density predicts pCR in bevacizumab treated patients

**Figure 1:**
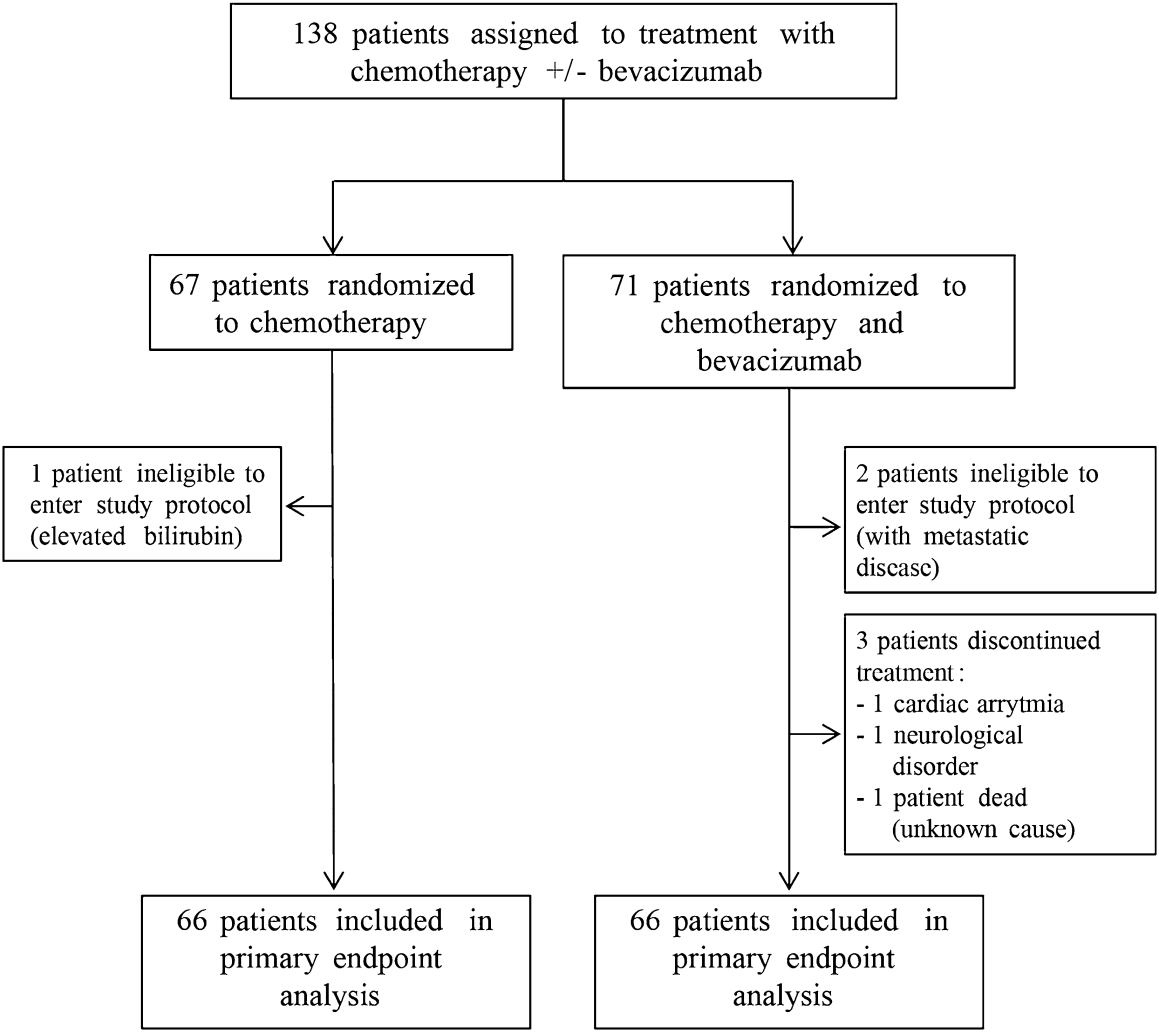
The NeoAva study design. Patients included in the study were randomized to receive chemotherapy with or without bevacizumab.

The NeoAva study design is presented in Fig. 1. As previously reported^27^, pCR in breast and axillary lymph nodes was seen in 15 patients (23%) in the bevacizumab-arm, and in 8 patients (12%) in the chemotherapy only-arm (Fisher’s exact test, *P* = 0.17). Among ER positive tumors, 11 patients (20%) responded in the bevacizumab-arm, compared with only 3 (5%) in the chemotherapy only-arm (Fisher’s exact test, *P* = 0.02). No significant difference in response was seen between the two treatment arms for ER negative patients.

**Figure 2:**
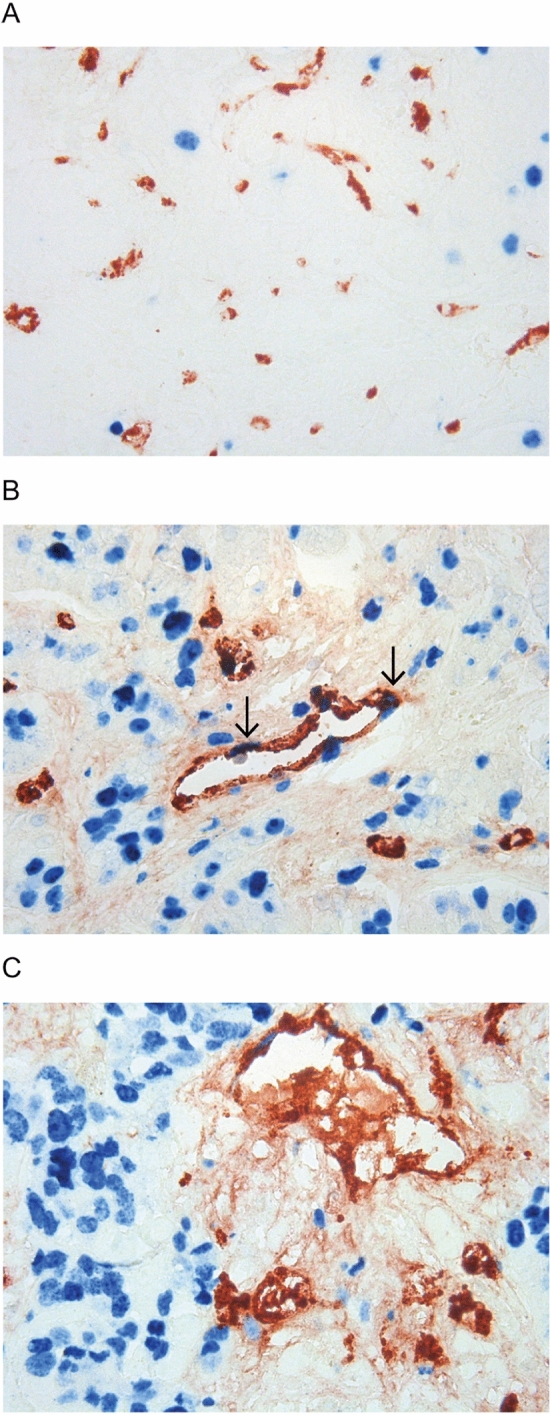
Factor VIII/Ki-67 dual immunohistochemical staining. Combined immunohistochemical staining of Factor VIII (red) and Ki-67 (blue), 400×. (**A**) Factor VIII positive, non-proliferative microvessels (MVD), (**B**) Proliferating microvessels (pMVD) by Factor VIII/Ki-67 co-expression, indicated by arrows, **C** Glomeruloid Microvascular Proliferation (GMP).

Microvessels were evaluated in 128 patients at baseline (64 in each treatment arm, four excluded) (Fig. 2). When selecting the three HPFs (high power fields) with the highest number of vessels, median MVD was 112.1 vessels/mm^2^ (mean 120.8, range 52.4–267.9), and median pMVD was 5.8 vessels/mm^2^ (mean 6.4, range 0.0–20.4). MVD and pMVD were dichotomized by median values, and no significant differences were seen between the two treatment arms at baseline (Pearson’s chi-square test).

**Figure 3:**
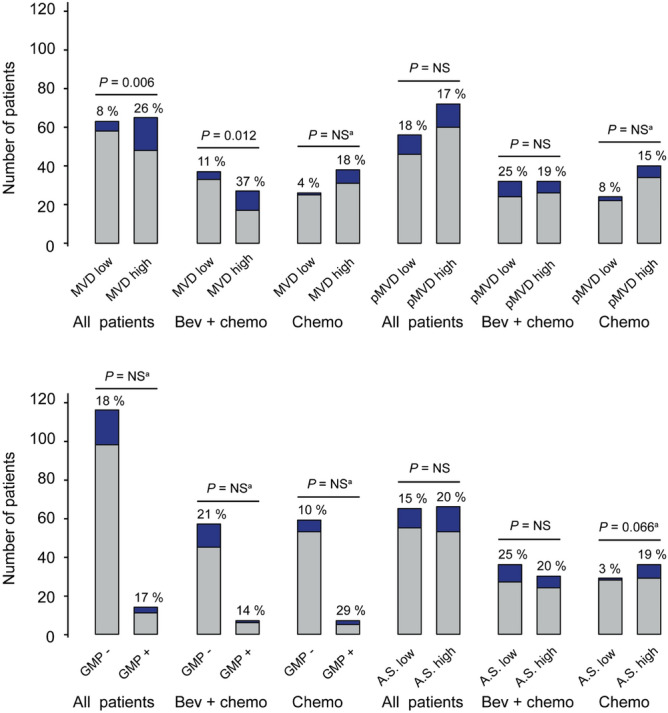
pCR according to MVD, pMVD, GMP and the 32-gene angiogenesis signature score. Each bar represents number of patients by high and low biomarker for all patients, the bevacizumab-arm (Bev + Chemo), and the chemotherapy only-arm (Chemo). pCR (pathologic complete response) rates are presented by %-estimates and indicated by dark blue color. *P* values by Pearson’s chi-square (χ^2^) test or Fisher’s exact test. ^a^Fisher’s exact test. *MVD* microvessel density, *pMVD* proliferative microvessel density, *A.S.* angiogenesis signature score. Median as cut-off value.

High MVD (≥ median) was significantly associated with pCR in the bevacizumab arm (OR, odds ratio, 4.9, *P* = 0.012), but not in the chemotherapy only-arm (Table 1 and Fig. 3). In the subgroup of patients treated with paclitaxel and bi-weekly bevacizumab, 14 of 45 patients responded^28^. High MVD was seen in 10 of the 14 responders in the paclitaxel subgroup (OR of 5.0; *P* = 0.018; 1 case with missing MVD-status). The subgroup receiving docetaxel and tri-weekly bevacizumab was too small for a similar comparison (1 of 21 patients responded). When analysing the subgroup of patients receiving paclitaxel in the chemotherapy only-arm, no association between MVD and pCR was found (n = 47, 6 responders, 1 case with missing MVD status). In contrast, pMVD was not associated with response in any of the treatment arms; this was also found for the paclitaxel subgroup (*P* = 0.7). At baseline, GMP was present in 14 of 130 patients evaluated (11%), seven in each arm, with no association with treatment response (Table 1 and Fig. 3). The 32-gene angiogenesis signature score was not significantly associated with response in any of the treatment arms (Fig. 3).

**Table 1:** MVD, pMVD, GMP according to pathologic complete response (pCR).

	MVD, N = 128					pMVD, N = 128					GMP, N = 130				
	≥ Median					≥ Median					Present				
	*N*	%	OR	95% CI	*P*	*N*	%	OR	95% CI	*P*	*N*	%	OR	95% CI	*P*
**All patients**															
*Pathologic response*															
Not complete	48	45	Ref			60	57	Ref			11	10	Ref		
Complete	17	77	4.1	1.4–12.0	.006	12	55	0.9	0.4–2.3	NS	3	14	1.5	0.4–5.9	NS^a^
**Bevacizumab-arm**															
*Pathologic response*															
Not complete	17	34	Ref			26	52	Ref			6	12	Ref		
Complete	10	71	4.9	1.3–17.8	.012	6	43	0.7	0.2–2.3	NS	1	8	0.6	0.1–5.7	NS^a^
**Chemotherapy only-arm**															
*Pathologic response*															
Not complete	31	55	Ref			34	61	Ref			5	9	Ref		
Complete	7	88	5.6	0.7–49.0	NS^a^	6	75	1.9	0.4–10.5	NS^a^	2	25	3.5	0.6–22.3	NS^a^

All patients: MVD and pMVD analyses: Pathologic complete response: *N* = 22; bevacizumab-arm: *N* = 14, chemotherapy only-arm, *N* = 8. GMP analyses: Pathologic complete response: *N* = 21; bevacizumab-arm: *N* = 13, chemotherapy only-arm, *N* = 8. MVD and pMVD; *N* = 128 (64 in each arm), GMP: *N* = 130 (bevacizumab-arm, *N* = 64, chemotherapy only-arm, *N* = 66).

*MVD* microvessel density, *pMVD* proliferative microvessel density, *GMP* glomeruloid microvascular proliferation, *N* number of patients, *OR* odds ratio, *CI* confidence interval, *P p* values by Pearson’s chi-square test.

^a^Fisher’s exact test.

Stratified by ER (estrogen receptor) status, high MVD tended to associate with response in the bevacizumab-arm in the ER positive subgroup. High MVD was seen in 7 of the 10 responders (70%) in ER-positive bevacizumab-treated patients, compared with high MVD seen in 15 of 42 (38%) non-responders (OR 4.2, *P* = 0.075, Fisher’s exact test; 2 cases with missing MVD-status). No significant associations were seen among ER negative cases, or in the chemotherapy only-arm. With the low number of patients included in these groups, and the few responders observed, these latter analyses should be interpreted with care. pMVD and GMP did not predict response in either arms when stratifying for ER status (data not shown).

### Angiogenesis markers pMVD, GMP and a 32-gene signature associate with aggressive tumor features

**Figure 4:**
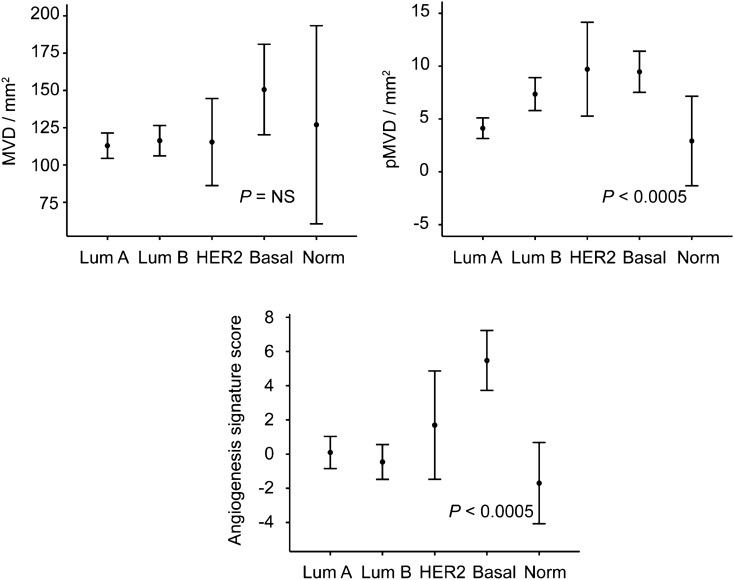
MVD, pMVD and the 32-gene angiogenesis signature score across intrinsic molecular breast cancer subtypes. MVD: microvessel density; pMVD: proliferative microvessel density, and the 32-gene angiogenesis signature score across intrinsic molecular breast cancer subtypes. Lum: luminal; HER2: HER2-enriched, Basal: basal-like; Norm: normal breast-like. *P*-values by the Kruskal–Wallis test, and error bars with 95% confidence interval of the mean. Number of patients; MVD and pMVD: *N* = 127 (Basal: *N* = 20, HER2: *N* = 9, Lum A: *N* = 53, Lum B: *N* = 40, Norm: *N* = 5), angiogenesis signature score: *N* = 131 (Basal: *N* = 21, HER2: *N* = 9, Lum A: *N* = 55, Lum B: *N* = 40, Norm: *N* = 6).

Increased vascular proliferation (pMVD) was significantly associated with high histologic grade (OR 2.6, *P* = 0.048), negative ER (OR 5.9, *P* = 0.003) and PR (progesterone receptor) status (OR 2.2, *P* = 0.032), and strongly associated with the triple negative phenotype (OR 9.0, *P* = 0.001) (Table 2). Further, pMVD differed between the intrinsic molecular subtypes (Kruskal–Wallis test, *P* < 0.0005) (Fig. 4), with high values seen significantly more often in the basal-like subgroup compared with other categories (OR 20.1, *P* = 0.001) (Table 2). Similar findings were seen for GMP, with a significant and strong association with high histologic grade (OR 6.2, *P* = 0.003), the triple negative phenotype (OR 3.7, *P* = 0.041), and a tendency to associate with ER negative tumors (OR 3.5, *P* = 0.051). Most GMP positive tumors (9 of 14 cases) had HER2-enriched or basal-like phenotypes, and the association with the basal-like group was highly significant when compared with the other subgroups (OR 5.4, *P* = 0.009).

**Table 2:** MVD, pMVD, GMP by clinico-pathologic variables and molecular subtypes of breast cancer.

	MVD, N = 128					pMVD, N = 128					GMP, N = 130				
	≥ Median					≥ Median					Present				
	*N*	%	OR	95% CI	*P*	*N*	%	OR	95% CI	*P*	*N*	%	OR	95% CI	*P*
**Estrogen receptor**															
Positive	53	50	Ref			54	51	Ref			9	8	Ref		
Negative	12	57	1.4	0.5–3.5	NS	18	86	5.9	1.6–21.2	0.003	5	24	3.5	1.0–11.7	0.051^a^
**Progesterone receptor**															
Positive	37	47	Ref			38	49	Ref			6	8	Ref		
Negative	28	56	1.4	0.7–2.9	NS	34	68	2.2	1.1–4.7	0.032	8	16	2.3	0.7–7.0	NS
**Triple negative**															
Absent	54	50	Ref			54	50	Ref			9	8	Ref		
Present	11	55	1.2	0.5–3.2	NS	18	90	9.0	2.0–40.7	0.001	5	25	3.7	1.1–12.7	0.041^a^
**Tumor stage**															
T2	14	37	Ref			18	47	Ref			6	16	Ref		
T3	47	59	2.4	1.1–5.4	0.026	46	58	1.5	0.7–3.3	NS	4	5	0.3	0.1–1.0	0.073^a^
**Histologic grade**															
1–2	45	48	Ref			49	53	Ref			6	6	Ref		
3	15	56	1.3	0.6–3.2	NS	20	74	2.6	1.0–6.6	0.048	8	30	6.2	1.9–19.9	0.003^a^
**Molecular subtype**															
Luminal A	25	47				20	38				2	4			
Luminal B	20	50				24	60				3	8			
HER2-enriched	3	33				6	67				3	33			
Basal-like	14	70				19	95				6	30			
Normal breast-like	2	40			NS^a^	2	40			< 0.0005^a^	0	0			0.004^a^
**Basal-like**															
Absent	50	47	Ref			52	49	Ref			8	7	Ref		
Present	14	70	2.7	1.0–7.4	0.056	19	95	20.1	2.6–156	< 0.0005	6	30	5.4	1.6–17.9	0.009^a^

Tumor stage: T4 tumors were excluded from the analyses. T2: 2,5- < 5 cm and T3: ≥ 5 cm in diameter. MVD and pMVD; *N* = 128, GMP; *N* = 130. Missing data: histologic grade: *N* = 8 (MVD and pMVD analyses) *N* = 7 (GMP analyses); molecular subtype: *N* = 1.

*MVD* microvessel density, *pMVD* proliferative microvessel density, *GMP* glomeruloid microvascular proliferation, *N* number of patients, *OR* odds ratio, *CI* confidence interval, *P p* values by Pearson’s chi-square test.

^a^Fisher’s exact test.

Whereas high MVD was associated with larger tumors (OR 2.4, *P* = 0.026, for T3 vs T2 tumors), and also significant for T3 and T4 tumors combined versus T2 (data not shown), there were no associations with any other clinico-pathologic features, including histologic grade, hormone receptor status (ER/PR), or the triple negative phenotype (Table 2). MVD was not associated with intrinsic molecular subtypes by PAM50 (Kruskal–Wallis, continuous; Fig. 4; or Pearson’s chi-square tests, dichotomous), although there was a trend for higher MVD in the basal-like group (OR 2.7, *P* = 0.056, Table 2).

The 32-gene angiogenesis signature score differed across molecular subtypes (*P* < 0.0005) (Fig. 4), with highest values in the basal-like subtype, and also high expression in the HER2-enriched category. The pattern was similar to the pMVD distribution (by immunohistochemistry) across intrinsic molecular subtypes, and the signature score was significantly correlated with pMVD values (Spearman’s ρ = 0.30, *P* = 0.001). Also, high signature score (by median) significantly associated with ER negative (OR 5.3, *P* = 0.002), triple negative (OR 4.9, *P* = 0.004) and basal-like tumors (OR 27.8, *P* < 0.0005), but not with PR status, tumor size or histologic grade (data not shown).

### Lower values of angiogenesis markers MVD and pMVD at week 12 but with no association to treatment response

Median MVD and pMVD were 53.9 and 1.5 vessels/mm^2^ in the bevacizumab-group at week 12, and 87.4 and 4.4 vessels/mm^2^ in the chemotherapy only-group. Using matched samples, MVD and pMVD showed significantly lower vessel numbers at week 12 compared with the values at baseline (in both arms) (*P* < 0.0005, Wilcoxon signed rank test). The average reduction in the bevacizumab-arm for MVD and pMVD was 51% and 61%, respectively, compared with 32% and 45% in the chemotherapy only-arm.

When dichotomizing MVD and pMVD vessel numbers at week 12, by baseline median values, only 1 of 38 patients in the bevacizumab-arm, and 6 of 39 patients in the chemotherapy only-arm, had high MVD, although this did not reach statistical significance (OR 6.7, CI 0.8–58.8, *P* = 0.11, Fisher’s exact test). For pMVD, 4 of 38 patients had pMVD above baseline median in the bevacizumab arm, and 18 of 39 in the chemotherapy only-arm (OR 7.3, CI 2.2–24.5, *P* = 0.001, Pearson’s chi-square test). At week 12, neither high MVD nor high pMVD (by baseline median values) were associated with response to treatment. pMVD at 12 weeks, but not MVD, still correlated with the 32-gene angiogenesis signature score estimated at 12 weeks (Spearman’s ρ = 0.34, *P* = 0.004); the angiogenesis score was not significantly different between the treatment arms at week 12 (data not shown).

The absolute difference in MVD and pMVD values from baseline to week 12 was calculated, and there was a trend towards patients in the bevacizumab-arm having a larger reduction in MVD (by median), than in the chemotherapy only-group (OR 2.3, CI 0.9–5.7, *P* = 0.082). For pMVD, this difference between baseline and week 12 was not significantly different between the treatment arms. Notably, the difference in MVD and pMVD values between baseline and week 12 were not significantly associated with response.

Neither MVD or pMVD at week 12, nor the difference from baseline to week 12 for MVD or pMVD, were associated with any clinico-pathologic variables or intrinsic breast cancer subtypes (Pearson’s chi-square or Fisher’s exact tests; data not shown).

## Discussion

In the neoadjuvant setting, improved pCR rates have been reported with the addition of bevacizumab to anthracycline and taxane based chemotherapy^8–12^. In the present study, we analysed whether tissue-based evaluation of angiogenesis markers MVD, pMVD, GMP and a 32-gene signature score could predict response to this treatment. Notably, high baseline MVD was the only marker that showed an association to increased pCR rates in the bevacizumab-arm. High MVD was still significantly associated with response when selecting for patients receiving bi-weekly bevacizumab in combination with paclitaxel (excluding patients receiving tri-weekly bevacizumab in combination with docetaxel). Also, in ER-positive bevacizumab-treated patients, there was a trend towards association with high MVD. Similar findings, with high MVD predicting response in breast cancer, were reported by Tolaney et al.^29^, but was not found in the smaller study by Yang et al.^30^. However, in these two studies, the chemotherapy regimens differed from ours, and subgroup analyses stratifying by ER status were not performed. High MVD and prediction of response to bevacizumab treatment was also reported in a recent study of ovarian cancer^31^.

We found that significantly more patients had lower pMVD values at week 12 (by baseline median) in the bevacizumab-arm compared with the chemotherapy only-arm. There was also a trend towards more patients in the bevacizumab-arm having a larger reduction in MVD from baseline to week 12. These findings are in line with results from preclinical models, were both pMVD and MVD were reduced after bevacizumab treatment^32^.

We initially hypothesized that patients with increased numbers of proliferating microvessels would respond better to anti-angiogenesis treatment. However, although pMVD was significantly lower in the bevacizumab-arm compared with the chemotherapy only-arm after 12 weeks of treatment, we found no association between pMVD values and response (pCR) to bevacizumab treatment in this study, neither using baseline values, week 12 values, nor the difference from baseline to week 12. However, baseline pMVD was significantly related to several markers of aggressive disease, including high histologic grade as well as the triple negative and basal-like phenotypes, whereas this was not seen for MVD. Further, the 32-gene angiogenesis signature score based on high pMVD levels in our previous study^24^, was not associated with response, but was correlated with tissue-based pMVD expression and showed similar associations as pMVD across intrinsic molecular breast cancer subtypes.

A limitation regarding analyses of tissue-based markers in the neoadjuvant setting is the small amount of tissue available. Core needle biopsies and not surgical specimens were examined, in contrast to previous studies in this field^18,20,24,25^, possibly influencing hot-spot selection for MVD and pMVD assessment^18,20,24^. Also, the frequency of GMP positive tumors was lower than previously reported^25^. However, despite the limited amount of tissue available, similar associations concerning clinico-pathologic variables and intrinsic molecular subtypes were found for pMVD and GMP as previously published^20,25,26,33^.

In summary, in this randomized phase II clinical trial of neoadjuvant chemotherapy ± bevacizumab in large or locally advanced HER2-negative primary breast cancer, high baseline MVD, reflecting the overall vascular density, was the only significant predictor of bevacizumab treatment response. Evaluation of MVD requires immunohistochemical staining of vessels on formalin-fixed paraffin-embedded tissues, a well-established method in pathology departments. Although there might be issues regarding reproducibility of vessel assessment, our experience is that high levels of agreement can be reached after initial training in the research setting, as supported by high inter- and intra-observer agreement in our study. However, whether MVD is a useful and robust clinical biomarker in relation to bevacizumab used as neoadjuvant therapy should be further validated in the routine setting as a potential basis for patient stratification. Other markers for activated angiogenesis, including pMVD, GMP, and a 32-gene signature score, did not predict response, but were all associated with aggressive breast cancer features including high histologic grade, basal-like and triple negative tumors.

## Methods

### Patients and study design

As described^27^, patients with large primary breast cancer (tumor diameter ≥ 2.5 cm) and locally advanced disease, previously untreated HER2-negative breast carcinomas were eligible. Other key inclusion criteria were WHO performance status 2, adequate hematologic and biochemical parameters, and no sign of metastatic disease. Additional prerequisites were normal organ function in general and normal left ventricular ejection fraction. Concomitant medications with anticoagulants, other than low dose acetylsalicylic acid (160 mg or lower) were not allowed. 138 patients were included during 2008–2012 (Oslo University Hospital, Oslo; St. Olav’s Hospital, Trondheim, Norway). Patients were stratified based on tumor size (2.5 ≤ T ≤ 5 cm, T > 5 cm) and hormone receptor status [positive for estrogen receptor (ER), progesterone receptor (PR), or both], and randomized 1:1 to receive chemotherapy ± bevacizumab. A block randomization procedure was used, and randomization was performed by the centralized research support facility at Oslo University Hospital (Oslo, Norway). The randomization list was not known to the personnel responsible for providing information or treatment to the patients. Six patients were excluded, leaving 66 patients in each arm for further analyses (Fig. 1). Clinical characteristics of included patients and adverse effects have been published^27^. Analyses performed in this study, focusing on angiogenesis markers and treatment response, were done as part of exploratory analyses and did therefore not form the basis for power calculations. Written informed consent was obtained from all patients prior to inclusion. The study was approved by the protocol review board at Clinic for Cancer Therapy at Oslo University Hospital, the regional ethics committee (REK SørØst) and the Norwegian Medicines Agency, and carried out in accordance with the Declaration of Helsinki, International Conference on Harmony/Good Clinical practice. Trial Registration: http://www.clinicaltrials.gov/; identifier NCT00773695. Registered on October 16, 2008.

### Treatment and response evaluation

The chemotherapy regimen consisted of four cycles of FEC100 (5FU 600 mg/m^2^, epirubicine 100 mg/m^2^, and cyclophosphamide 600 mg/m^2^) given every third week, followed by four cycles with docetaxel (100 mg/m^2^) every third week or 12 weekly infusions with paclitaxel 80 mg/m^2^. Bevacizumab was administered intravenously at a dose of 15 mg/kg every third week, or 10 mg/kg every other week in patients receiving docetaxel (n = 21) or paclitaxel (n = 45), respectively^28^.

The primary end-point was pathologic complete response (pCR), defined as the absence of invasive carcinoma in the breast and in the axillary lymph nodes.

### Immunohistochemistry

Formalin-fixed paraffin-embedded routine core needle biopsies at time of diagnosis, in addition to a biopsy collected before treatment start and at week 12, were available. Of the 132 patients in the cohort, 131 and 94 patients had available tissue at time of diagnosis, and at week 12, respectively.

Staining was done on 4–5 µm formalin-fixed paraffin-embedded standard tissue sections. Pre-treatment of the sections was done, i.e. pressure cooker antigen retrieval in Target Retrieval Solution pH 6.0 (Dako S1699, Glostrup, Denmark), and 8 min incubation with Dual Endogenous Enzyme-Blocking Reagent (Dako 2003). The sections were incubated for 60 min at room temperature with polyclonal rabbit anti-human Factor VIII related antibody (Dako A0082) diluted 1:1600 and monoclonal mouse Ki-67 antibody (Dako M7240) diluted 1:200 in Antibody diluent (Dako S3022). Alkaline phosphatase goat anti-mouse (H + L) IgG (Southern Biotech, Birmingham, AL, USA) diluted 1:100 in horseradish peroxidase goat anti-rabbit EnVision (Dako K4011) was applied for 30 min at room temperature. Visualization of Ki-67 with Ferangi Blue™ Chromogen System (Biocare Medical, Concord, CA, USA, FB812S) for 15 min and of Factor VIII with AEC + Substrate-Chromogen (Dako K3469) for 15 min at room temperature. Counterstaining was not done. Positive and negative controls were included in each staining round.

### Evaluation of tissue-based angiogenesis markers

#### Microvessel density (MVD)

As described by Weidner et al.^18^, the tissue sections were first examined at low magnifications (25× and 100×) to identify the most vascular areas of the invasive tumor (“hot-spots”) within the core needle biopsies. Then, vessels were counted in 3–10 consecutive fields, depending on amount of available tissue, at high magnification (400x, field size 0.23 mm^2^)^34^.

Vessels were recognized by their Factor VIII positive endothelial cells combined with their morphology. Any red-stained endothelial cell or cluster of endothelial cells that was clearly separate from adjacent microvessels, tumor cells, and other connective tissue elements, was considered a single, countable vessel. Notably, vessel lumens were not necessary for a structure to be defined as a vessel, but in cases with GMP or long, winding and branching vessels, each lumen was regarded as a single vascular unit and counted. The fields used to count vessels should contain at least 50% invasive tumor tissue (with exceptions for the week 12 biopsies, as discussed below), and vessels associated with necrotic areas or fibrotic scars were excluded^34^.

#### Proliferative microvessel density (pMVD)

pMVD is a measure of the number of microvessels containing proliferating endothelial cells within the evaluated tumor area. Proliferating endothelial cells were recognized by their co-expression of Factor VIII and Ki-67. The same microscopic fields and vessel criteria were used for MVD and pMVD assessment. Ki-67 positive nuclei outside the endothelial cell layer or within the vessel lumen were not counted. Microvessels were regarded as proliferating when they contained one or more proliferating endothelial cells^34^.

#### Evaluation of tissue biopsies at week 12

Biopsies from 94 patients were available at week 12. Of these, 46 patients had biopsies with ≥ 50% tumor tissue in at least three high power fields (HPFs) and 12 patients had < 50% tumor cells in at least three HPFs in their biopsy. In biopsies from 19 patients, no tumor cells were seen, but there were changes in the connective tissue consistent with chemotherapy induced tissue reaction. Vessels were counted in these biopsies. However, if the biopsy did not include tumor cells or tissue changes suggesting chemotherapy treatment reactions, patients were excluded because of uncertainty regarding the representativeness of the sample (*N* = 12). Also, five cases had less than three HPFs for evaluation and were excluded, leaving 77 patients for analyses (38 in the bevacizumab-arm and 39 in the chemotherapy only-arm).

#### Glomeruloid microvascular proliferation (GMP)

Focal glomerulus-like aggregates of closely associated and multilayered Factor VIII positive endothelial cells, GMPs, were evaluated (by O.S.), as either present or absent^25^. Lumen formation was not necessary for the glomerulus-like structures to be registered as GMP. At baseline, 130 patients had enough tumor tissue for GMP evaluation.

#### Variables and cut-off values

For MVD and pMVD, the three HPFs with highest number of vessels were selected for further analysis. At baseline, four patients had less than three HPFs for evaluation of MVD and pMVD, and were therefore excluded, leaving 128 patients, 64 in each treatment arm, for further analyses. Baseline MVD and pMVD were dichotomized by the median value. Cut-off by the upper quartile was also explored, but gave somewhat weaker results (data not shown). For MVD and pMVD at week 12, the baseline median values were used as cut-off values. Vascular proliferation Index (VPI), the ratio between the number of microvessels containing proliferating endothelial cells and the total number of Factor-VIII positive microvessels (pMVD/MVD), given as a percentage, was also analysed but gave somewhat weaker results (data not shown). The average value of all HPFs, and the one HPF with the highest vessel count for MVD, pMVD, and VPI, were also examined, but the results were similar or weaker as for the maximum of three HPFs (data not shown).

#### Inter- and intra-observer agreement

Before evaluating MVD, pMVD and VPI in the research series, training was done on a test set of 24 tissue sections (endometrial carcinoma). These were scored separately by an experienced pathologist (I.M.S.). Difficulties concerning evaluation of vessels were discussed. Inter- and intra-observer agreements were estimated by Kappa (к) and Spearman’s rho (ρ), dichotomized by median value, and the training was not ended until both inter- and intra-observer agreements reached a satisfactory and stable value. The final inter- and intra-observer agreement reached Kappa values (κ) ≥ 0.7 and Spearman’s rho (ρ) > 0.8. Intra-observer agreement for the main study, for both MVD and pMVD showed Kappa values, κ = 0.7 and Spearman’s ρ = 0.9^34^.

### Gene expression analysis

Gene expression profiling was performed using 40 ng total RNA and one color Sureprint G3 Human GE 8 × 60 k Microarrays (Agilent Technologies), as previously described^27^. Molecular subtype determination was performed using the PAM50 algoritm^35^. A 32-gene expression signature based on differentially expressed genes in pMVD high vs low cases (by median value), originally determined in an endometrial cancer cohort^24^, was applied in the present data set. The angiogenesis signature score was generated by subtracting the sum of the expression values for the down-regulated genes from the sum of expression values for the up-regulated genes. The microarray data are available in the ArrayExpress database (http://www.ebi.ac.uk/arrayexpress) under accession number E-MTAB-4439.

### Statistical analysis

Data were analysed using SPSS (version 22.0, IBM corp., Armonk, NY, USA). Associations between categorical variables were evaluated by Pearson’s chi-square (χ^2^) test or Fisher’s exact test when appropriate. Odds ratios (OR) and 95% confidence intervals (CI) are presented. For paired data (baseline and week 12 biopsies), Wilcoxon signed rank test was used. Spearman’s rank correlation test was applied when comparing bivariate continuous variables, and the Kruskal–Wallis test for analysing differences across intrinsic breast cancer subtypes.

All statistical tests were two-sided, and statistical significance was assessed at 5% level, and *P* values between 5 and 10% were regarded as borderline significant.

### Ethical approval and informed consent

The study was approved by the protocol review board at Clinic for Cancer Therapy at Oslo University Hospital, the regional ethics committee (REK SørØst) and the Norwegian Medicines Agency, and carried out in accordance with the Declaration of Helsinki, International Conference on Harmony/Good Clinical practice. Written informed consents were obtained from all patients prior to inclusion.

## Data Availability

The datasets generated and analysed during the current study are available from the corresponding author upon reasonable request. The microarray dataset supporting the conclusions of this article is available in the ArrayExpress database, accession number E-MTAB-4439 (http://www.ebi.ac.uk/arrayexpress). The trial is registered in the http://www.clinicaltrials.gov/ website; identifier NCT00773695. Registered on October 16, 2008.
